# An antigen-specific immunotherapeutic, AKS-107, deletes insulin-specific B cells and prevents murine autoimmune diabetes

**DOI:** 10.3389/fimmu.2024.1367514

**Published:** 2024-03-07

**Authors:** David G. Alleva, Andrea R. Delpero, Thillainaygam Sathiyaseelan, Sylaja Murikipudi, Thomas M. Lancaster, Mark A. Atkinson, Clive H. Wasserfall, Liping Yu, Ramya Ragupathy, Rachel H. Bonami, Todd C. Zion

**Affiliations:** ^1^ Department of Pharmacology, Akston Biosciences, Inc., Beverly, MA, United States; ^2^ Departments of Pathology, Immunology, and Laboratory Medicine, College of Medicine, and Diabetes Institute, The University of Florida, Gainesville, FL, United States; ^3^ Barbara Davis Center for Diabetes, School of Medicine, University of Colorado, Aurora, CO, United States; ^4^ Division of Rheumatology and Immunology, Department of Medicine, Vanderbilt University Medical Center, Nashville, TN, United States; ^5^ Department of Pathology, Microbiology, and Immunology, Vanderbilt University Medical Center, Nashville, TN, United States; ^6^ Vanderbilt Center for Immunobiology, Vanderbilt University Medical Center, Nashville, TN, United States; ^7^ Vanderbilt Institute for Infection, Immunology, and Inflammation, Vanderbilt University Medical Center, Nashville, TN, United States

**Keywords:** autoreactive B cell, autoimmunity, type 1 diabetes, Fc-fusion protein, antigen specific immunotherapeutic, insulin autoantigen, insulin

## Abstract

**Introduction:**

The antigen-presenting cell function of insulin-reactive B cells promotes type 1 diabetes (T1D) in non-obese diabetic (NOD) mice by stimulating pathogenic T cells leading to destruction of insulin-producing β-cells of pancreatic islets.

**Methods/Results:**

To target insulin-reactive B cells, AKS-107, a human IgG1 Fc molecule fused with human insulin A and B chains, was engineered to retain conformational insulin epitopes that bound mouse and human B cell receptors but prevented binding to the insulin metabolic receptor. AKS-107 Fc-mediated deletion of insulin-reactive B cells was demonstrated *via ex vivo* and *in vivo* experiments with insulin-reactive B cell receptor transgenic mouse strains, VH125Tg/NOD and Tg125(H+L)/NOD. As an additional immune tolerance feature, the Y16A mutation of the insulin B_(9-23)_ dominant T cell epitope was engineered into AKS-107 to suppress activation of insulin-specific T cells. In mice and non-human primates, AKS-107 was well-tolerated, non-immunogenic, did not cause hypoglycemia even at high doses, and showed an expectedly protracted pharmacokinetic profile. AKS-107 reproducibly prevented spontaneous diabetes from developing in NOD and VH125Tg/NOD mice that persisted for months after cessation of treatment, demonstrating durable immune tolerance.

**Discussion:**

These preclinical outcomes position AKS-107 for clinical development in T1D prevention settings.

## 1 Introduction

Type 1 Diabetes (T1D) is driven by dysregulated islet autoantigen-specific B and T lymphocytes that escape control of immune tolerance mechanisms and mediate damage of insulin-producing beta cells in pancreatic islets (1, 2). The pre-diabetes *Stages 1 and 2* of T1D manifest with autoreactive B and T lymphocyte reactions to several islet autoantigens that promote the underlying pathology leading to overt diabetes, *Stage 3* (3). Insulin appears to be a dominant autoantigen in many T1D subjects [reviewed in (4)]. In fact, a hallmark of early subclinical disease processes is the pronounced high titers of insulin autoantibodies (IAA) produced by insulin-reactive B cells (4–7). Previous studies implicate insulin-reactive B cells in human T1D as antigen-presenting cells (APC) that promote autoreactive CD4+ and CD8+ T cell activation, based on immunophenotyping of peripheral blood and pancreatic islets of deceased T1D subjects of all ages, especially those < 7 years (2, 8–17). The inappropriate APC function of insulin-reactive B cells appears, in part, to be caused by defective B cell tolerance mechanisms in T1D subjects in which peripheral autoreactive B cells are not of a usual anergic state (11, 12, 18). Moreover, the pathogenic role of B cells is supported by prevention or suppression of diabetes via therapeutic treatments that broadly target B cells in NOD mice [i.e., mAbs to CD20, CD22, and BlyS/BAFF; reviewed in (19–21)] and in human clinical trials [i.e., anti-CD20 mAb, rituximab (22–24)].

This APC function of B cells has been thoroughly studied in NOD mouse models of T1D [reviewed in (25)]. Notably, NOD mice genetically engineered for deficiency of mature B cells do not develop diabetes, and this lack of disease progression is associated with dramatic reductions in islet antigen-specific CD4^+^ and CD8^+^ T cells in secondary lymphoid organs and insulitic lesions. Furthermore, B cell depletion in NOD mice led to expansion of T_reg_ cells able to suppress islet antigen-specific pathogenic T cells, suggesting B cells directly affect pathogenic T cells (20, 26). NOD mouse B cells genetically modified to be MHC class I or II deficient (to prevent antigen presentation to CD8+ or CD4+ T cells) prevented diabetes (27–30). Evidence that insulin-reactive B cells play a central role in T1D development was demonstrated by diabetes development in 125mAb-transgenic NOD mice in which most (Tg125(H+L)/NOD) or part (VH125Tg/NOD) of the B cell receptor (BCR) repertoire is insulin-reactive, but not in mice that lacked anti-insulin B cells (i.e., VH281Tg/NOD) (31, 32). Both female and male VH125Tg/NOD mice have an accelerated and more highly penetrant diabetes incidence relative to the standard female (and male) NOD mice that is mediated by IFN-γ-producing insulin-specific T cells driven by the APC action of insulin-reactive B cells (31–35).

Considering the APC function of insulin-reactive B cells, in addition to the proof-of-concept therapeutic outcomes of broadly targeting the B cell compartment in both mouse and human T1D, we designed a novel antigen-specific immunotherapy (ASI), AKS-107, that specifically targets insulin-reactive B cells for deletion. AKS-107 comprises a metabolically inactivated form of insulin expressed as a human IgG1 Fc-fusion molecule that does not bind the metabolic insulin receptor but maintains binding to polyclonal insulin-reactive B cells in T1D subjects. Upon binding BCRs, the Fc moiety of the ASI is designed to mediate antibody-dependent cellular cytotoxicity (ADCC) of the B cell. In addition, the dominant T cell epitope, insulin B chain 9-23 (B_9-23_), was modified at the tyrosine 16 amino acid residue position to an alanine (Y16A) which is known to prevent activation of pathogenic T_eff_ in NOD mice and human T1D subjects (36–38). Herein, we present the pharmacology of AKS-107 that demonstrates specific targeting of insulin-reactive B cells and efficacy in preventing diabetes in NOD and VH125Tg/NOD mice. These results suggest that AKS-107 could be administered early in the pre-diabetes process at the first signs of the IAA titer biomarker, which is a rational approach to restore and maintain immune tolerance and prevent T1D as suggested by others (39).

## 2 Materials and methods

### 2.1 Animals

Animal studies were performed at Akston Biosciences, Inc. (Beverly, MA) and the University of Florida (Gainesville, FL). NOD (*NOD/ShiLtJ*), BALB/c, and VH125Tg/NOD (*NOD.Cg-Tg(Igh-6/Igh-V125)2Jwt/JwtJ*) mice were purchased from The Jackson Laboratory (Bar Harbor, ME). The Tg125(H+L)/NOD mouse strain is heterozygous for both the heavy and light Ig chain transgenes of 125mAb derived by crossing VH125Tg/NOD (125mAb mu H chain gene) with Vk125Tg (125mAb kappa L chain gene, NOD.Cg-Tg(Igk-C/Igk-V125)1Jwt/JwtJ) mice and were produced and provided by Dr. James W. Thomas (Department of Medicine, Vanderbilt University, Nashville, TN) (31, 32). All animals were maintained on autoclaved food and water and housed under specific pathogen-free conditions, and experimentation was approved by the respective Institutional Animal Care and Use Committees. All non-human primate (NHP; i.e., cynomolgus monkey) studies were performed with protocols approved by the Animal Care and Use Committee at Biomere (Worcester, MA) in accordance with the National Institutes of Health guide for the care and use of laboratory animals (NIH Publications No. 8023, revised 1978), and with veterinary care in accordance with the testing facility standard operating procedures and regulations outlined in the applicable sections of the Final Rules of the Animal Welfare Act regulations (9 CFR).

### 2.2 Fc fusion protein molecule constructs and preparation

**Figure 1:**
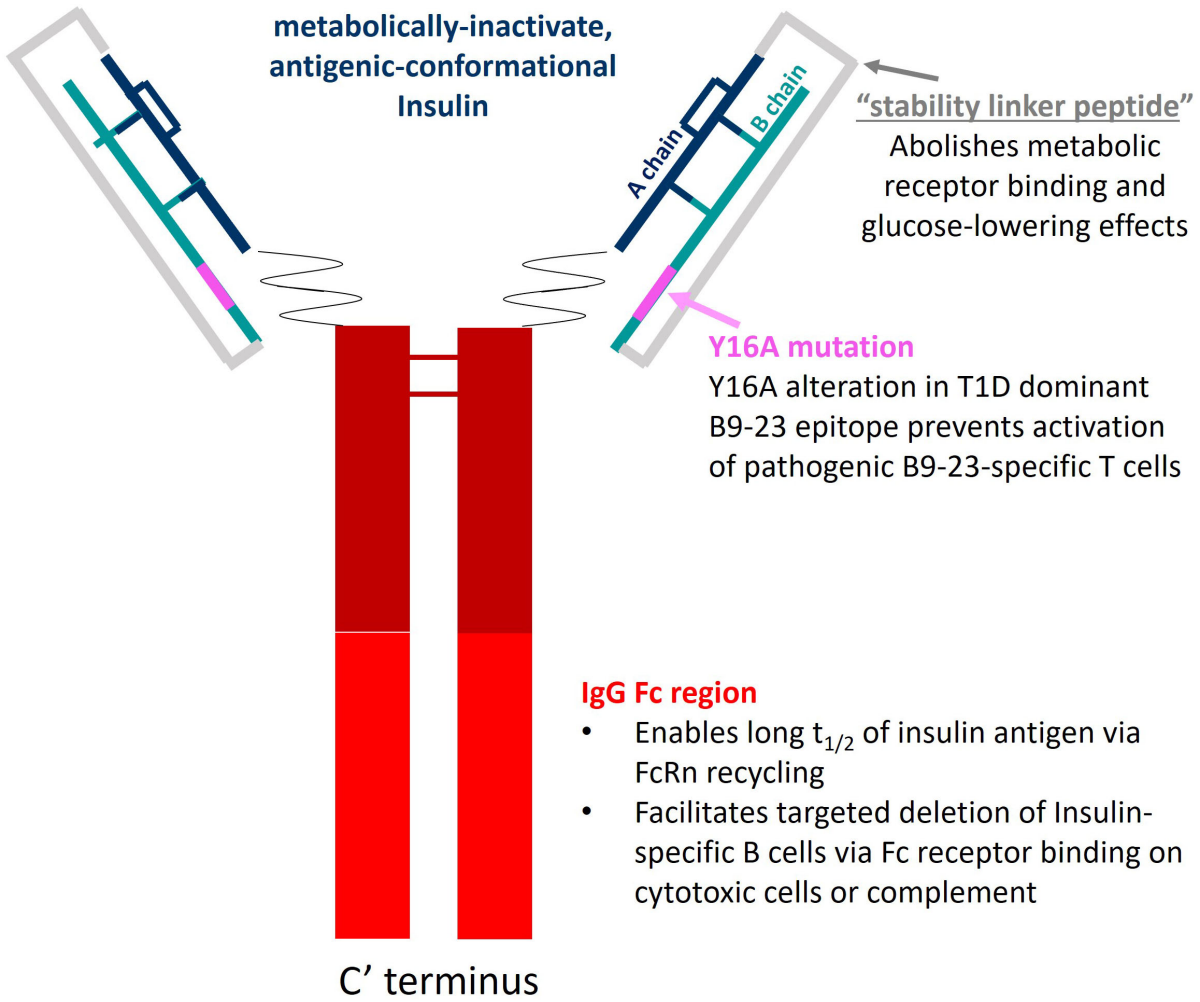
AKS-107 schematic. AKS-107 is a human IgG1 Fc fusion protein containing an inactive human insulin analog engineered to avoid binding the insulin metabolic receptor but to retain conformational insulin antigenic epitopes that bind mouse and human anti-insulin B cell receptors to delete insulin-reactive B cells.

AKS-107 is a recombinant fusion protein comprising the complete human insulin A chain sequence connected to a three amino acid linker sequence connected to the complete human insulin B chain (containing an alanine substitution at the tyrosine 16 position; Y16A) that is connected to a human IgG1 Fc fragment containing a portion of the hinge region fused to the full CH2 and CH3 domains, all encoded by a single nucleic acid molecule expressed in CHO-K1 cells (**Figure 1**). The AKS-130 Fc-fusion insulin analog is a unique construct that retains metabolic activity via the insulin receptor (see **Supplementary Table S1** for the respective Fc-fusion molecule amino acid sequences). All Fc-fusion proteins were expressed in a CHO-K1 cell line derivative (see **Supplemental Methods** for details).

### 2.3 AKS-107 binding potency to insulin-specific antibodies

Conformational integrity of the insulin analog moiety of AKS-107 was assessed via binding to two mouse anti-insulin mAbs (i.e., 125mAb capture/123mAb detection, which were purified from hybridomas and were a gift from Dr. James Thomas) via ELISA (see **Supplemental Methods**). The binding affinity of AKS-107 to circulating human serum IAAs was measured *via* a competitive inhibition ^125^I-labeled rhINS radioimmunoassay [(40) and see **Supplemental Methods**].

### 2.4 Insulin receptor binding and activation

Insulin binding to naturally expressed insulin receptors on human IM-9 cells (ATTC# CCL-159) was determined via a competitive inhibition binding flow cytometry assay with phycoerythrin (PE)-labeled rhINS (see **Supplemental Methods**). The insulin receptor activation assay consisted of U2OS cells genetically engineered to express the human insulin receptor (PathHunter^®^ Insulin Bioassay kit, Eurofins/DiscoverX, Fremont, CA) that were incubated with different concentrations of rhINS (positive control), AKS-130 (active insulin-Fc analog positive control), AKS-107, or human IgG mAb (Fc negative control) and kinase activity was assessed by the addition of chemiluminescence substrate (PathHunter Detection reagent cocktail) using the PerkinElmer Envision™ instrument (see **Supplemental Methods**).

### 2.5 Cytotoxicity assay of murine insulin-reactive B cells

Tg125(H+L)/NOD splenocytes (>95% insulin-binding B cells of the B lymphocyte compartment) and macrophages were isolated as previously described (32, 41) in which splenocytes (5x10^5^ cells) and macrophages (2.5x10^4^ cells) were cultured in culture medium (IMDM with 10% fetal bovine serum) in wells of sterile round-bottom 96-well tissue culture plates (Thermo Fisher Scientific Inc, Waltham, MA) with or without dilutions of AKS-107 for 72 h (200 µL total volume), washed 3 times to remove AKS-107, and cultured in 200 µL of culture medium for an additional 24 h to allow for BCR turnover. Cells were then transferred to V-bottom microtiter plates, washed once with cold MACS buffer (Miltenyi Biotech, Bergisch Gladbach, Germany), resuspended in FACS staining medium (HBSS/0.1% Sodium Azide/4% horse serum) containing rhINS-biotin/streptavidin-labeled Microbeads (Miltenyi Biotech, #130-048-101) plus Alexa Fluor^®^ 488-labelled B220 mAb, and incubated on ice for 30 min. Cells were washed twice with cold-MACS buffer, resuspended in FACS staining buffer containing APC-labelled anti-µBead mAb for 20 minutes on ice, washed once, and fixed with 2% paraformaldehyde for flow cytometric analysis (FACSCalibur, Becton Dickinson, Franklin Lakes, NJ). AKS-107 concentration-dependent killing of insulin^+^ B cells was calculated as the percentage of insulin^+^ B cells from control cultures in which EC50 and EC90 values were calculated using the non-linear *[Agonist]* vs. *response-Variable slope (four parameters)* algorithm in GraphPad Prism 10.1 software.

### 2.6 *In vivo* pharmacokinetics in mice

BALB/c, NOD, VH125Tg/NOD, and Tg125(H+L)/NOD mice were weighed and blood samples were obtained via submandibular vein venipuncture collected into BD Microtainer Capillary Blood Collector tubes (Thermo Fisher), allowed to clot at room temperature for 30 min, centrifuged at 8,000 rpm for 8 minutes to obtain serum, and stored at -20°C. Blood glucose via tail bleed was measured with an ACCU-CHEK Advantage glucometer (Roche Diagnostics, Indianapolis, IN). The next day, animals received a single *i.p.* or *s.c*. injection of AKS-107 or AKS-130 and blood samples were collected periodically to obtain serum. Serum concentrations of AKS-107 and AKS-130 were measured via sandwich ELISA using a mouse anti-insulin Ab (Abcam, #D6C4) as the capture reagent (1:50 dilution in PBS/0.1% Tween-20/10% Superblock/20%Horse Serum buffer; all products from Thermo Fisher) and horseradish peroxidase-conjugated goat anti-human IgG Fc Ab (Bethyl Laboratories/Fortis Life Sciences, Waltham, MA, #A80-148A) as the detection Ab (1:30,000 diluted in PBST/SB/HS buffer), in addition to the tetramethylbenzidine (TMB) solution (Mabtech, Cincinnati, OH) and 1% H_2_SO_4_ stop reagent. Standard curves were constructed using known amounts of AKS-107 spiked in PBST/SB/HS buffer containing normal mouse serum at the same percentage as test serum samples (e.g. 2-5% serum in PBST/SB/HS buffer). Absorbance per well was measured at 450 nm via a microplate reader to generate optical density (OD)_450_ values that were proportional to AKS-107 serum concentration determined via interpolation to the standard curve using *Softmax Pro* 7.0 software (Molecular Devices, San Jose, CA). *GraphPad Prism 10.1* software was used to calculate C_max_, t_max_, AUC, and terminal phase elimination rate (half-life; t_1/2_).

### 2.7 *In vivo* pharmacodynamics in mice

Insulin-reactive B cells were enriched and quantified from blood and spleens of VH125Tg/NOD mice treated with AKS-107 or vehicle control. Single-cell suspensions in FACS staining buffer were treated with FITC-labelled anti-B220 mAb, PE/Cy7-labelled anti-IgM, and rhINS-conjugated magnetic µBeads (MaxFlex Microbead Kit, Miltenyi Biotech) and incubated on ice for 30 min. Splenocyte subpopulations were analyzed via staining with BV421-labelled anti-CD21 mAb, BV510-labelled anti-CD23 mAb, and PE-labelled anti-CD43 mAb added at the same time as anti-B220, anti-IgM, and the µBeads. Cells were washed with MACS buffer (HBSS/2mM EDTA/0.5% Horse serum/0.1% Azide) and labeled with anti-µBead-APC mAb for 20 min on ice. After washing and fixing, the cell suspension was applied to a Miltenyi MS magnetic column and flow-through material was collected to determine the total percentage of B cells in each sample. Columns were then washed twice with MACS buffer after which bound cells were eluted into tubes, centrifuged, and re-suspended in a buffer containing standard counting beads followed by FACS analysis. B220^+^ Insulin BCR^+^ B cells and respective subpopulations in both splenocytes and blood cells were analyzed with a BD LSR-II flow cytometer (BD Biosciences, San Diego, CA).

### 2.8 Spontaneous diabetes monitoring

Six to 8 week-old male and female VH125Tg/NOD mice and 4 week-old female NOD mice received weekly or bi-weekly doses of AKS-107 or vehicle (saline) via 50 µL *i.p*. or *s.c*. (abdominal flank) injection routes. Fasting blood glucose (FBG) was monitored bi-weekly via tail bleed and measured with an ACCU-CHEK Advantage glucometer (Roche Diagnostics, Indianapolis, IN). Diabetes was diagnosed when FBG was >240 mg/dL on 2 occasions at least 7 days apart. This FBG cut-off was derived from 11 prior experiments at the Akston lab from the mean ± 3xSD of FBG levels of non-diabetic NOD mice (unpublished observation). FBG measurements from all experiments were obtained in a blinded fashion.

## 3 Results

### 3.1 AKS-107 IgG1 Fc-fusion insulin analog maintains conformational epitopes but is not metabolically active

**Figure 2:**
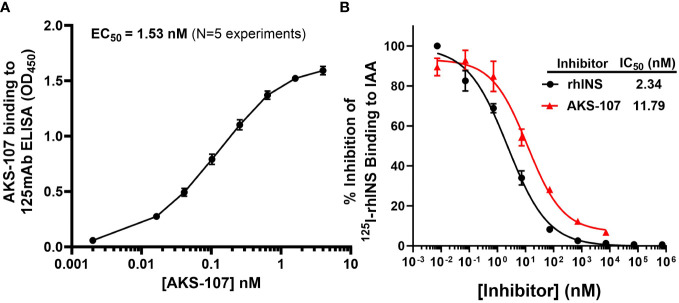
AKS-107 binding to insulin-specific Abs. The potency of AKS-107 to bind anti-insulin Abs was demonstrated with mAbs **(A)** and with polyclonal Abs in pooled sera from newly diagnosed T1D patients with IAAs before exogenous insulin treatment **(B)**. The anti-insulin mAb, 125mAb, was used to capture AKS-107 and another insulin-specific mAb, 123mAb, was used to detect bound AKS-107 via ELISA (data represent the mean ± SEM of 5 experiments) **(A)**. For binding to polyclonal Abs, IAA+ pooled serum was incubated overnight with 125I-rhINS with and without unlabeled rhINS or AKS-107 in triplicate, and bound 125I-rhINS was precipitated via protein-A/G Sepharose and detected via radioimmunoassay (data are mean ± SEM) **(B)**. The concentration response curves were used to calculate the Effective Concentration 50% (EC_50_) or the IC_50_ via log(agonist) vs. response – Variable slope (four parameters) algorithm of GraphPad Prism 10.1 software.

**Figure 3:**
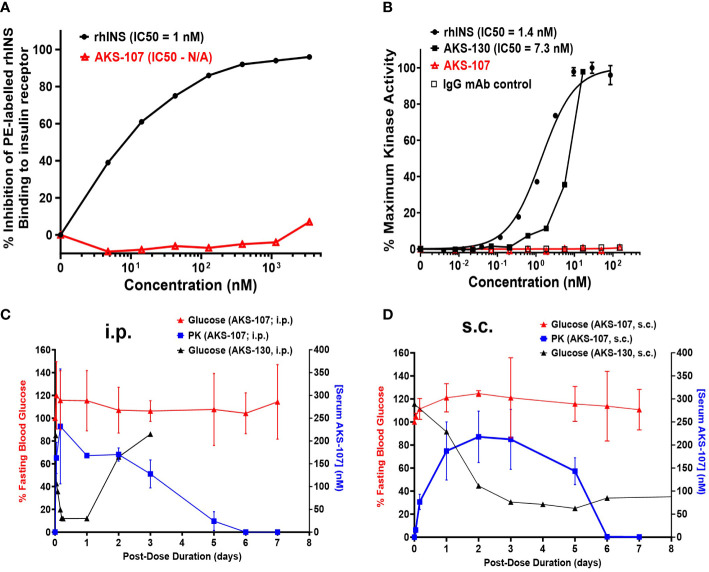
AKS-107 does not bind or activate the human or mouse insulin metabolic receptor. **(A)** Insulin receptor binding: human IM-9 cells that naturally express the human insulin metabolic receptor were treated with different concentrations of AKS-107 or rhINS in the presence of 0.5 µg/mL PE-rhINS and fixed with paraformaldehyde prior to flow cytometric analysis. PE-rhINS binding was calculated as MFI/sample in which 100% binding were cells bound with PE-rhINS in the absence of AKS-107 or cold rhINS. IC_50_ values were calculated using the non-linear 4-parameter log[Inhibitor] vs. response-Variable slope (four parameters) algorithm in GraphPad Prism 10.1 software (data represent one of two experiments that had similar results). **(B)** Insulin receptor phosphorylation: The U2OS cells genetically engineered to express the human insulin receptor (PathHunter^®^ Insulin Bioassay, Eurofins/DiscoverX) were incubated with different concentrations of rh-INS (positive control), AKS-130 (active insulin-Fc IgG control), AKS-107, and human IgG mAb (negative control) and insulin receptor activation was assessed via activated kinase conversion of a chemiluminescence substrate (PathHunter Detection Reagent) (data represent one of two experiments that had similar results). IC_50_ values were calculated using GraphPad Prism 10.1 software. The lack of insulin receptor activation by AKS-107 was determined *in vivo* in BALB/c mice after a single *i.p.* **(C)** or *s.c.* **(D)** injection of AKS-107 or AKS-130 via measurement of FBG levels over 7 days, with assessment of serum levels of AKS-107 via ELISA (n = 3 mice per group).

To specifically target polyclonal insulin-reactive B cells, we developed a fusion protein, AKS-107, comprised of a genetically engineered protein sequence containing the human insulin A chain linked to a three amino acid linker peptide linked to the human insulin B chain linked to a human IgG1 Fc sequence (**Figure 1**). The presence of the specific three amino acid linker peptide was empirically derived (data not shown) to create a structurally rigid insulin molecule incapable of binding the metabolic insulin receptor thus avoiding glycemic modulation. Conformational insulin epitopes in AKS-107 relevant to anti-insulin BCR binding were confirmed via ELISA in which AKS-107 bound the insulin-specific mAb, 125mAb, derived from a T1D pathogenic insulin-specific BCR and a second anti-insulin mAb, 123mAb (32, 42) (**Figure 2A**). AKS-107 also bound human polyclonal IAAs in pooled sera (from IAA^+^ patients with newly diagnosed T1D before exogenous insulin treatment) *via* an ^125^I-rhINS competitive radioimmunoassay, demonstrating an affinity similar to that of unlabeled rhINS (**Figure 2B**). AKS-107 could not bind (**Figure 3A**) or activate (**Figure 3B**) the human insulin metabolic receptor naturally expressed on the insulin-sensitive EBV-transformed B lymphoblastoid cell line, IM-9. In addition, a single dose of AKS-107 (2 mg/kg = 31 nmol/kg) administered *via i.p.* (**Figure 3C**) or *s.c.* (**Figure 3D**) injection did not suppress FBG levels in BALB/c mice during a 7-day period despite its substantial and persistent levels in blood during the first 5 days. As a positive control, the metabolically active human insulin-Fc analog, AKS-130, strongly reduced FBG levels within hours after *i.p*. administration that were restored after 2 to 3 days (**Figure 3C**). Similar effects, but with delayed kinetics, were demonstrated as expected upon *s.c.* administration of AKS-130 (**Figure 3D**). Similarly, administration of AKS-107 (0.4 mg/kg, *i.v.*) to cynomolgus monkeys had no effect on FBG after one (**Supplementary Figure S1A**; AKS-107 terminal phase elimination rate, t_1/2_ = 6.9 days) and three (**Supplementary Figure S1B**) doses.

### 3.2 AKS-107 mediates targeted depletion of insulin-reactive B cells *in vitro*

**Figure 4:**
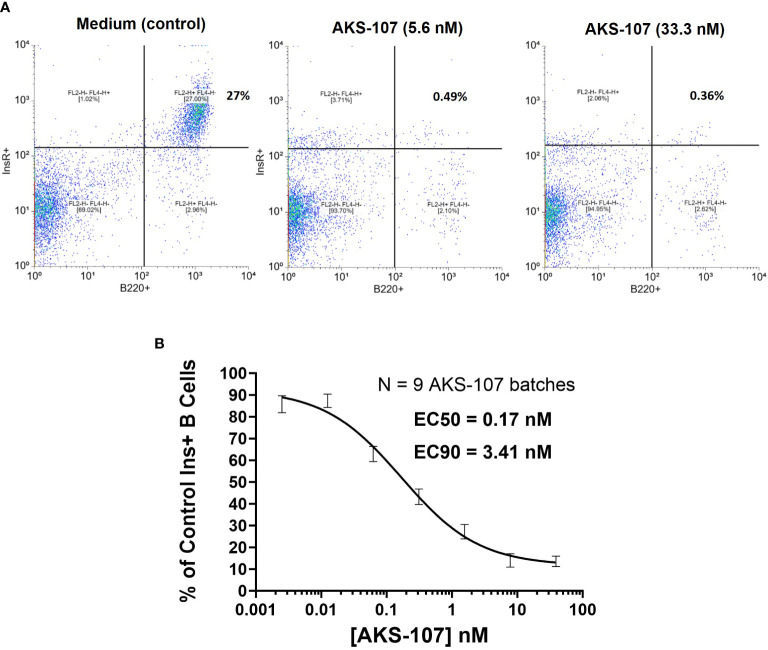
AKS-107 mediates cytotoxicity of insulin-specific murine B cells. Co-culture of Tg125(H+L)/NOD splenocytes (5x10^5^ cells/well) and macrophages (2.5x10^4^cells/well) were cultured with or without AKS-107 at 1:5 serial dilutions for 72 h, washed to remove AKS-107, and cultured for an additional 24 h to allow for B cell receptor turnover. After washing, cells were incubated with rhINS-conjugated microbeads (APC-labeled) and Alexa Fluor^®^ 488-labelled B220 mAb for 30 min on ice, washed and fixed with 2% paraformaldehyde prior to flowcytometric analysis via FACSCalibur. **(A)** A representative flowcytometry analysis of Insulin+ B cells (B220+) from Control (medium) and AKS-107-treated cultures. **(B)** A concentration-dependent killing of Insulin+ B cells by AKS-107 calculated as the percentage of Control Insulin+ B cells. Data represent a single experiment testing 9 independently produced batches of AKS-107 and the mean ± SEM are presented. EC50 and EC90 values were calculated using the non-linear 4-parameter [Agonist] vs. response-Variable slope (four parameters) algorithm in GraphPad Prism 10.1 software.

To assess whether AKS-107 could mediate ADCC of insulin-reactive B cells, AKS-107 was incubated with Tg125(H+L)/NOD mouse splenocytes [source of >95% insulin-reactive B cells (34)] and FcR-expressing cytotoxic macrophages, and insulin-reactive B cells were measured via flow cytometry. AKS-107 reduced the insulin-reactive B cell (i.e., rhINS^+^ B220^+^) population in a dose-dependent manner with an EC_50_ of 0.17 nM and EC_90_ of 3.4 nM (**Figure 4C**). In contrast, the non-insulin specific B220^+^ B cell population was unaffected (**Figures 4A, B**). Integrity of the Fc moiety of AKS-107 was confirmed upon binding to human IgG Fc receptors (FcR), FcRI, FcRIIa, FcRIIb, FcRIIIa, and FcRIIIb, consistent with ADCC function, and to complement (C1q), consistent with the potential for complement-dependent cytotoxicity (**Supplementary Figure S2**). Note that the NOD genetic background lacks a functional C5 gene (43) which nullifies any contribution that complement-dependent cytotoxicity may have had in AKS-107-mediated B cell depletion in studies presented here.

### 3.3 *In vivo* PK and PD profiles of AKS-107

**Figure 5:**
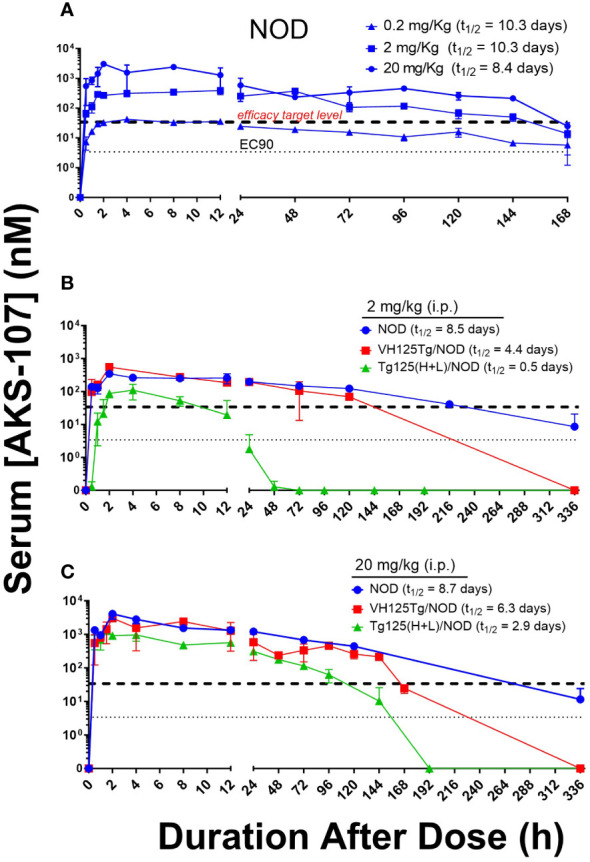
AKS-107 PK profiles in different insulin-specific BCR genetic NOD mouse backgrounds. A single i.p. injection of AKS-107 at indicated dose levels was administered to female NOD mice (0.2, 2, and 20 mg/kg, N=3 per timepoint; experiment #1) **(A)** and NOD, VH125Tg/NOD, or Tg125(H+L)/NOD mice (2 and 20 mg/kg, N=3 per timepoint; experiment #2) **(B, C)** and serum levels of AKS-107 (via ELISA) were measured at indicated times. AKS-107 serum t_1/2_ (terminal phase elimination rate) was calculated via the Exponential-One Phase Decay algorithm in GraphPad Prism 10.1 software.

To evaluate the *in vivo* insulin-reactive B cell cytotoxic potency of AKS-107, PK profiles of AKS-107 were obtained in NOD, VH125Tg/NOD, and Tg125(H+L)/NOD mice to derive dosing strategies. NOD mice were initially used to compare PK profiles among three AKS-107 dose levels, 0.2, 2 and 20 mg/kg, in which a single *i.p.* dose of each dose level was administered, and AKS-107 serum levels were determined at different times over 7 days (i.e., 168 hours; **Figure 5A**). The maximum concentration of AKS-107 in blood (C_max_) was achieved at approximately 2 h after dosing for all dose levels in which a dose-dependent relationship was apparent and maintained for 6 days (i.e., 144 h). Considering the AKS-107 EC_90_ value of 3.4 nM derived from the *in vitro* cytotoxicity experiments (see **Figure 4**), a 10-fold greater “*efficacy target level*” (i.e., 34 nM) was used as an estimate to ensure an optimal dosing strategy for PD and efficacy experiments. While the AKS-107 C_max_ of the 0.2 mg/kg dose exactly reached the *efficacy target level* during the first 24 h, the Cmax of the 2 and 20 mg/kg groups were approximately 10- and 100-fold greater than the *efficacy target level*, respectively, and AKS-107 serum levels induced by 2 and 20 mg/kg were maintained above this target value for 6 days (**Figure 5A**). The PK profile of the 2 mg/kg NOD group was consistent with that of the 2 mg/kg BALB/c PK profile in **Figure 3C**. Therefore, 2 mg/kg (**Figure 5B**) and 20 mg/kg (**Figure 5C**) were evaluated in a separate experiment with the three mouse strains that reflected differences in insulin-reactive B cell content as a percentage of the total B cell compartment; i.e., Tg125(H+L)/NOD (>95%) >> VH125Tg/NOD (~ 3%) > NOD (<0.1%) (31, 32, 34). Administration of a single *i.p.* dose of AKS-107 resulted in expected C_max_ and kinetic profiles of AKS-107 levels based on differences in dose level (i.e., within each strain, AKS-107 C_max_ and duration were greater in 20 vs. 2 mg/kg groups) and among strains showing an inverse correlation between AKS-107 levels and endogenous insulin-reactive B cell content (i.e., Tg125(H+L)/NOD showed the lowest C_max_ and fastest clearance vs. VH125Tg/NOD vs. NOD). These results also define a dose level range between 0.2 and 2 mg/kg for multiple dosing regimens for PD and efficacy studies, as the higher AKS-107 serum levels of the 20 mg/kg dose level did not appear to provide an advantage relative to 2 mg/kg.

**Figure 6:**
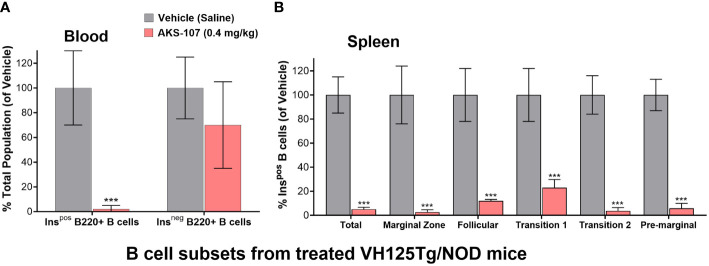
AKS-107-mediates *in vivo* reduction of insulin-specific B cells in VH125Tg/NOD mice. Four i.p. injections of Vehicle or AKS-107 (0.4 mg/kg) were administered to female VH125Tg/NOD mice (N=5 mice per group) twice weekly for 14 days after which insulin-stained B cells (B220+) in blood **(A)** and spleen **(B)** were measured via flow cytometry using labelled rhINS and B220-specific mAb. Different B cell subpopulations in splenocyte preparations were labeled with the mAbs specific to the following surface markers; Total splenic B cells (B220+), Marginal Zone (CD21hi/CD23low), Follicular (IgM+/CD21+/CD23+), Transitional 1 (CD21low/CD23low), Transitional 2 (IgMhi/CD21+), and Pre-marginal (IgMhi/CD21hi) **(B)**. The mean % Ins+ B cell percentages of the Vehicle control group of the indicated cell populations were used as the denominator for each respective mouse sample (Vehicle and AKS-107 treated groups) to derive the mean ± SEM of each dosing group. ***p<0.001, one-tailed student’s t-test (GraphPad Prism 10.1).

The *in vivo* capacity of AKS-107 to specifically target insulin-reactive B cells was determined using VH125Tg/NOD mice because this strain has elevated insulin-reactive B cell frequencies measurable via flow cytometry that correlate with exacerbated spontaneous diabetes onset and frequency, but retaining the non-insulin-binding B cell repertoire (31). VH125Tg/NOD mice were treated with AKS-107 or vehicle control via *i.p.* injection of 0.4 mg/kg twice weekly for 2 weeks (i.e., 4 doses) after which blood and spleens were analyzed for insulin-reactive and non-insulin-reactive B cell content (**Figure 6**). AKS-107 treatment almost completely removed the peripheral insulin-reactive B cell population while preserving the non-insulin-binding B cell population in blood (**Figure 6A**) as well as splenic marginal zone, follicular, T1, T2, and pre-marginal zone B cell sub-populations (**Figure 6B**).

### 3.4 AKS-107 reduced diabetes incidence in T1D-prone mice

Male VH125Tg/NOD mice were favored as a proof-of-concept model for evaluating AKS-107 efficacy because i) the reduction in insulin-reactive B cells can be readily measured and therefore translated to efficacy in VH125Tg/NOD mice, ii) diabetes onset and incidence is accelerated in VH125Tg/NOD mice such that male mice actually acquire diabetes with similar incidence to the standard female NOD model [whereas < 20% of male NOD mice typically acquire diabetes; our unpublished observation and (44)], thus associating insulin-reactive B cells directly with disease in males [note that female VH125Tg/NOD show extremely aggressive disease kinetics and incidence relative to the males (31)], and iii) unlike NOD mice, VH125Tg/NOD mice do not develop anti-insulin Ab responses, thus avoiding the potential of human epitopes in AKS-107 to induce cross-reactive anti-mouse insulin neutralizing Abs.

**Figure 7:**
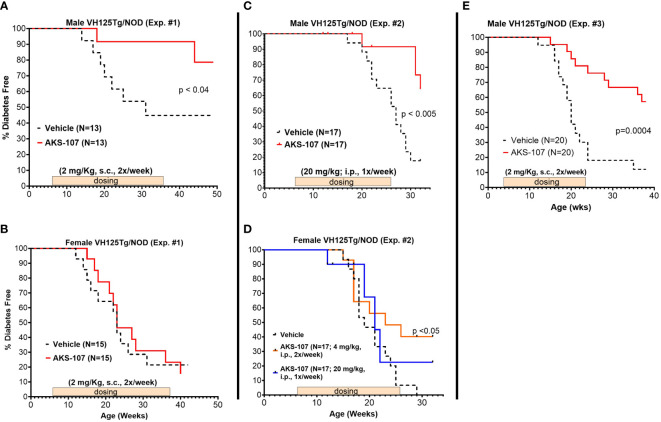
AKS-107 prevention of T1D in VH125Tg/NOD mice. Six to 8 week-old male **(A, C, E)** and female **(B, D)** VH125Tg/NOD mice received AKS-107 injections at the indicated frequencies, dose levels, routes of administrations (*i.p.* or *s.c.*), and durations in three different experiments. FBG levels were monitored weekly for the onset of diabetes defined as >240 mg/dL on two measurements 7 days apart. Diabetes incidence is presented as the percentage of mice free of diabetes per dosing group. Survival curve statistical comparison between Vehicle- and AKS-107-treated groups was performed via the Log-rank (Mantel-Cox) test in GraphPad Prism 10.1.

Three efficacy experiments were performed in young VH125Tg/NOD male and female mice (6- to 8-weeks of age) that received AKS-107 or vehicle once or twice per week for different durations (**Figure 7**). In the first experiment in which AKS-107 was administered *s.c.* at 2 mg/kg twice weekly for 30 weeks, diabetes incidence in male cohorts receiving vehicle was first observed at 14 weeks of age reaching a maximum of 55% at 32 through 50 weeks (end of study), whereas diabetes incidence in the AKS-107-treated group reached only 10% at 18 through 44 weeks and ended at a maximum of 20% at 50 weeks, thus demonstrating significant disease protection by AKS-107 in male VH125Tg/NOD mice (**Figure 7A**), but not against the highly aggressive disease in the female mice (**Figure 7B**). The second experiment explored AKS-107 administered *i.p.* at higher dose levels of 20 mg/kg once weekly in males and females, and 4 mg/kg twice weekly only in females, for 20 weeks (**Figures 7C, D**). AKS-107 showed an efficacy profile in male mice similar to the first experiment (**Figure 7C**) whereas modest but significant protection was demonstrated in female cohorts at the 4 mg/kg twice weekly dosing, but not at 20 mg/kg once weekly dosing (**Figure 7D**) (or 2 mg/kg twice weekly, **Figure 7B**), suggesting that both increased dosing frequency and dose level are required to suppress the very aggressive disease course in female VH125Tg/NOD mice. In the third experiment, AKS-107 was administered *s.c.* 2 mg/kg twice weekly to male mice for 20 weeks (**Figure 7E**) (versus 30 weeks in Experiment 1 via the same dosing regimen, **Figure 7A**) which also demonstrated significant disease protection. Collectively, these results demonstrate that AKS-107 administered either *s.c.* or *i.p.* significantly prevented the spontaneous incidence of diabetes in male VH125Tg/NOD mice at dose levels consistent with the PD results of B cell depletion, and that higher doses of AKS-107 can reduce diabetes incidence in the highly aggressive female VH125Tg/NOD model.

**Figure 8:**
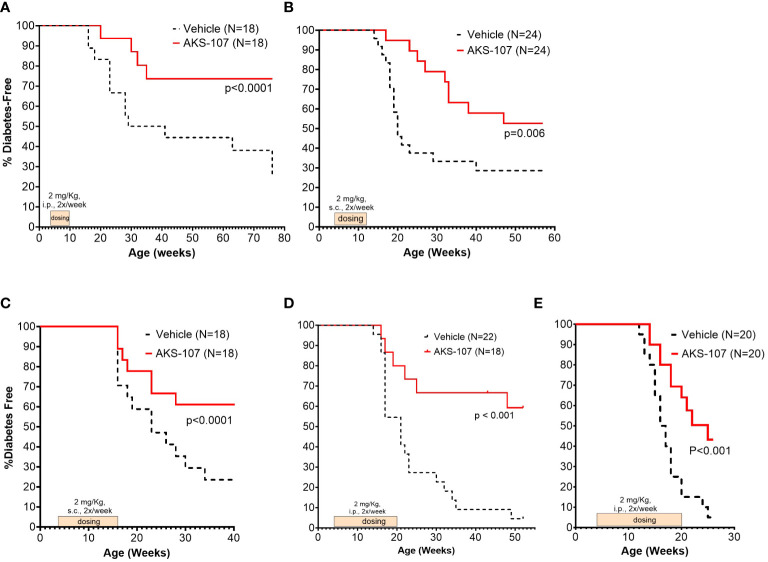
Limited courses of AKS-107 lead to sustained T1D protection in female NOD mice. Four to 6 week-old female NOD mice received AKS-107 injections of 2 mg/kg or Vehicle twice weekly at the indicated routes of administrations (*i.p.* or *s.c.*) and durations in five different experiments **(A–E)** and FBG levels were monitored weekly for the onset of diabetes defined as >240 mg/dL on two measurements 7 days apart. Diabetes incidence is presented as the percentage of mice free of diabetes per dosing group, with the number of mice per group indicated on each plot. Survival curve statistical comparison between Vehicle- and AKS-107-treated groups was performed via the Log-rank (Mantel-Cox) test in GraphPad Prism 10.1.

**Figure 9:**
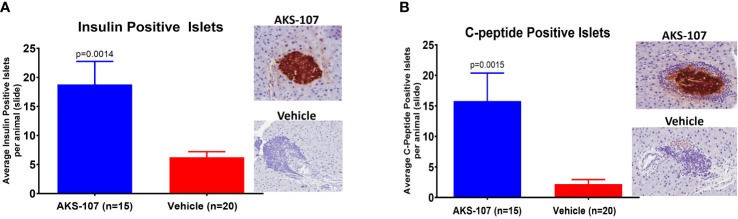
Preservation of pancreatic islet insulin-producing beta cells from female NOD mice treated with AKS-107. Four to 6 week-old female NOD mice received i.p. injections of Vehicle or AKS-107 at 2 mg/kg twice weekly for 16 weeks (see **Figure 8E** for diabetes incidence) in which mice were sacrificed at 26 weeks of age for insulin- and C-peptide-specific cell staining via immunohistochemistry analyses of whole pancreas samples. Briefly, whole pancreata were harvested, formalin fixed, embedded, and sectioned such that longitudinal slices of pancreas were placed on slides. Sections were stained for insulin using a rat anti-insulin primary Ab (R & D systems, #MAB1417) and a donkey anti-rat IgG secondary Ab conjugated to Alexa488, and for leukocyte infiltration (insulitis) using Hematoxylin. Slides were scanned on an Aperio whole slide scanner (20x magnification), and then analyzed in a blinded fashion. One slide was analyzed per animal and for each animal the number of insulin islets per slide were counted. Mean insulin **(A)** and C-peptide **(B)** positive islets per slide per animal are shown (p, student’s t-test) beside representative images of positive (AKS-107-treated) and negative (Vehicle-treated) islet samples.

These results suggest AKS-107 treatment may result in immune tolerance defined by the maintenance of efficacy well after halting dosing (see **Figures 7A, C, E**). Because immune tolerance mechanisms (involving both T and B cells) have been well-established in female NOD mice (45), we tested whether relatively brief periods of AKS-107 dosing of young NOD mice would induce long-term efficacy after dosing cessation. In a series of five experiments with 4 week-old female mice, AKS-107 was delivered in the first two experiments via *i.p.* (**Figure 8A**) or *s.c*. (**Figure 8B**) at 2 mg/kg for 6 weeks or 8 weeks, respectively, resulting in significant reductions in disease incidence out to 76 weeks of age (25% AKS-107 vs. 75% Vehicle incidence; **Figure 8A**) and 57 weeks of age (48% AKS-107 vs. 71% Vehicle incidence; **Figure 8B**). Extending the dosing period out to 12 weeks (**Figure 8C**) and 16 weeks (**Figures 8D, E**) also showed significant maintenance of disease suppression, demonstrating a potential immune tolerance effect that persisted well after the expected insulin-reactive B cell repopulation in the periphery following AKS-107 cessation. To ensure therapeutic robustness, the experiment represented in **Figure 8E** was performed at a different laboratory (i.e., Mark Atkinson lab, University of Florida, Gainesville, FL), in which immunohistochemistry analysis of whole pancreas samples obtained at the end of the 26-week study demonstrated that AKS-107 treatment preserved approximately 4-fold greater numbers of insulin^+^ (and C-peptide^+^) islets than vehicle control (**Figure 9**).

## 4 Discussion

Here, we engineered an insulin-Fc analog as a unique ASI that avoided binding to and activation of the insulin metabolic receptor while maintaining conformal integrity to bind and delete insulin-reactive B cells (via ADCC) that was associated with diabetes prevention in mice via an immune tolerance mechanism. Others created a metabolically inactive insulin analog molecule as an immune tolerance ASI (e.g., B25-Asp); however, the initial efficacy study in NOD mice demonstrating prevention of disease (46) was not reproducible (47). While both B25-Asp and AKS-107 ASI’s contain insulin modification that foster a tolerogenic APC-T cell interaction (including the Y16A mutation in AKS-107), the Fc moiety of AKS-107 offers the additional mechanism of directly depleting pathogenic B cell APCs and extending the t_1/2_ to increase bioexposure. We confirmed the function of the Y16A mutation in AKS-107 by its suppression of a B_(9–23)_-specific TCR hybridoma (*unpublished observation*). AKS-107 exhibited consistent positive efficacy outcomes in NOD and VH125Tg/NOD mice among multiple studies and between two laboratories.

The established role of insulin-reactive B cells as a dominant APC in driving disease in the VH125Tg/NOD mouse was exploited in defining the therapeutic target and efficacy of AKS-107. Relevant to the design of AKS-107, the Thomas lab performed proof-of-concept studies with 123mAb that binds endogenous insulin bound to the insulin-reactive 125-BCR (34, 42). While 123mAb selectively deleted insulin-reactive B cells in VH125Tg/NOD mice, it only prevented diabetes in female NOD but not VH125Tg/NOD mice (male mice were not evaluated) (34). It was suggested that 123mAb treatment failed to protect female VH125Tg/NOD mice because of the artificial continual *de novo* generation of overwhelming numbers of insulin-reactive B cells from bone marrow relative to the very low numbers in NOD females (34). These results are consistent with the modest efficacy of AKS-107 in preventing disease in female VH125Tg/NOD mice only after increasing the dosing regimen to 4 mg/kg, twice weekly (vs. 2 mg/kg, twice weekly or 20 mg/kg, once weekly). Commercial applicability of the 123mAb approach is limited by its inability to deplete anti-insulin B cells expressing mAb123-like BCRs. In fact, 123mAb liability is evident when a single 4 mg/kg dose was administered *i.p*. to BALB/c mice that dramatically elevated serum insulin concentrations (i.e., >250-fold higher than normal levels; *unpublished observation*). A F(ab’)_2_ version of mAb123 therapy had less robust depletion of mature anti-insulin B cells in the periphery than full-length mAb123, implicating ADCC as a dominant mechanism of mature anti-insulin B cell depletion (42).

There were several limitations to the current study. First, insulin-specific B cells in blood, spleen, and pancreas exist at frequencies too rare for accurate detection in the NOD mouse strain, which precluded their assessment in non-transgenic NOD mice. For this reason, we emphasize that VH125Tg/NOD mice were used to make the correlation between insulin-specific B cell modulation and efficacy to suppress diabetes by AKS-107 because significant insulin-specific B cells in blood and spleen exist that can be accurately measured and are known to drive the pronounced disease incidence in male mice. Accordingly, AKS-107 treatment efficiently deleted insulin-specific B cells after a 2-week treatment in addition to preventing diabetes in these mice. Second, while analyses of pancreatic islet immune cell types (including regulatory phenotypes) would be helpful in understanding the mechanisms of immune tolerance induced by AKS-107, our efficacy studies were not designed to address such immunological questions because separate cohorts would be needed to obtain pancreata samplings in a terminal fashion prior to diabetes onset. That is, pancreatic sampling and analyses after diabetes onset or at the end of such efficacy studies would obviously be confounded by islet “survivor bias”, particularly in the control group, which would confound comparisons of proinflammatory and regulatory lymphocytic infiltrates (that may have been present in islets prior to their destruction). However, such efforts evaluating B and T cell phenotypes in immune organs and pancreas in parallel cohorts via terminal analyses during the course of disease development are planned for future studies. Third, a human IgG1 isotype control mAb was not included in our studies because immune responses (Ab and T cell) to unique epitopes contained in the entire mAb (including those of the CDRs) would inevitably be different from those against AKS-107, and thus potentially confound interpretation of disease outcomes after AKS-107 treatment. That is, any modulation of disease in the mAb isotype “control” treated cohort would not necessarily reflect a similar modulation due to AKS-107 treatment.

Of note, in prior studies we detected low/modest anti-human Fc Ab titers [*i.e.*, anti-drug Abs (ADAs)] in some, but not all, NOD mice after several weeks of dosing (unpublished observation). For this reason, we limited dosing durations in female NOD mice, which surprisingly, allowed us to demonstrate an immune tolerance effect (*i.e.*, maintained efficacy after dosing stopped). Because of the possibility of ADAs developing in an unpredictable fashion, we aggressively dosed twice per week to maintain sufficient drug levels to counter any possible additional drug clearance caused by ADAs that may have developed. Note also that the most significant risk in dosing mice to determine efficacy with a human Fc fusion protein is not necessarily development of anti-Fc Ab titers but, rather, the risk of inducing “anti-insulin titers” that would clearly have a direct impact on the mechanism of action of AKS-107. For this reason, VH125/NOD mice were also used because anti-insulin Abs do not develop in these mice.

The preclinical efficacy data in mice and preliminary PK and safety data in NHP warrant development of AKS-107 as a clinical candidate for preventing T1D in high-risk prediabetic adults and children identified via islet autoantibody (iAb) screening (3, 48). The long preclinical period occurring after initial iAb detection offers an opportunity to therapeutically intervene prior to overt clinical diabetes (49). Moreover, the antigen-specific function of AKS-107 may work synergistically in combination with the recently FDA-approved anti-CD3 mAb therapeutic, teplizumab, if administered during *Stage 2*, for prevention or delay of *Stage 3* T1D (50, 51). Indeed, a recent clinical study in pediatric T1D subjects demonstrated positive outcomes upon treatment with T_reg_ cell therapy followed by CD20^+^ B cell depletion therapy (i.e., rituximab) (23). AKS-107 is positioned for clinical safety and efficacy studies as a mono- or combination-therapy intended to prevent progression to overt diabetes.

## Data availability statement

All relevant data is contained within this article, and the original contributions presented in this study are included in the article/**Supplementary Material**, and that further inquiries can be directed to the corresponding author.

## Ethics statement

The animal studies were approved by Animal Care and Use Committees (IACUCs) at Akston Biosciences, Inc. and at the Departments of Pathology and Pediatrics, The University of Florida. The studies were conducted in accordance with the local legislation and institutional requirements. Written informed consent was obtained from the owners for the participation of their animals in this study.

## Author contributions

DA: Conceptualization, Data curation, Formal analysis, Investigation, Methodology, Supervision, Writing – original draft, Writing – review & editing. AD: Formal analysis, Investigation, Methodology, Writing – review & editing. TS: Conceptualization, Data curation, Formal analysis, Investigation, Methodology, Supervision, Writing – review & editing. SM: Conceptualization, Data curation, Formal analysis, Investigation, Methodology, Supervision, Writing – review & editing. TL: Conceptualization, Formal analysis, Investigation, Methodology, Supervision, Writing – review & editing. MA: Conceptualization, Data curation, Formal analysis, Investigation, Methodology, Supervision, Writing – review & editing. CW: Conceptualization, Data curation, Investigation, Methodology, Supervision, Writing – review & editing. LY: Data curation, Investigation, Methodology, Supervision, Writing – review & editing. RR: Investigation, Methodology, Writing – review & editing. RB: Conceptualization, Writing – review & editing. TZ: Conceptualization, Formal analysis, Funding acquisition, Investigation, Resources, Supervision, Writing – review & editing.
