# Supplementary material for: An antigen-specific immunotherapeutic, AKS-107, deletes insulin-specific B cells and prevents murine autoimmune diabetes
Source: Front Immunol. 2024 Mar 7;15:1367514. doi: 10.3389/fimmu.2024.1367514 (PMC10954819; doi:10.3389/fimmu.2024.1367514)
Supplement: Supplementary file 1 [file DataSheet_1.docx]

**Supplemental Methods for the manuscript entitled:**

***An antigen-specific immunotherapeutic, AKS-107, deletes insulin-specific B cells and prevents murine autoimmune diabetes*** *by David G. Alleva, Andrea R. Delpero, Thillainaygam Sathiyaseelan, Sylaja Murikipudi, Thomas M. Lancaster, Mark A. Atkinson, Clive H. Wasserfall, Liping Yu, Ramya Ragupathy, Rachel H. Bonami, and Todd C. Zion.*

**Contents**

[1) Production, characterization, and formulation of insulin analog Fc fusion proteins 1](#_Toc158210493)

[2) AKS-107 binding potency to insulin-specific antibodies 3](#_Toc158210494)

[3) AKS-107 binding affinity to insulin receptors expressed on IM-9 cells 4](#_Toc158210495)

[4) Insulin receptor phosphorylation 5](#_Toc158210496)

[5) Cytotoxicity assay of murine insulin-reactive B cells 6](#_Toc158210497)

[6) In vivo pharmacokinetics and pharmacodynamics in non-human primates (NHPs) 6](#_Toc158210498)

# Production, characterization, and formulation of insulin analog Fc fusion proteins

AKS-107 and AKS-130 Fc-fusion proteins were expressed in a Chinese Hamster Ovary (CHO) cell line, CHO-K1, in which endogenous glutamine synthetase (GS) genes were knocked-out *via* recombinant technology prior to transduction of target genes with proprietary stable, high-expression mammalian DNA vectors optimized for CHO expression and GS selection (LakePharma, Belmont, CA). The sequence of each construct was confirmed prior to initiating scale up experiments. These suspension-adapted CHO cells were cultured in a humidified 5% CO_2_ incubator at 37 °C in a chemically defined media that did not contain serum or other animal-derived products (CD OptiCHO; Thermo Scientific, Waltham, MA). Approximately 80 million of these suspension-adapted CHO cells growing in exponential growth phase were transfected by electroporation using MaxCyte® STX® system (MaxCyte, Inc., Gaithersburg, MD) with 80 µg DNA to a create a stable CHO cell line for AKS-107 or AKS-130 human IgG-Fc fusion proteins. After 24 hrs, transfected cells were enumerated and seeded into CD OptiCHO selection medium containing between 0-100 µM methionine sulfoximine (MSX) at a cell density of 0.5 x 10^6^ cells/mL in a shaker flask and incubated at 37 ºC with 5% CO_2_. During this selection process, cells were centrifuged and resuspended in fresh selection media every 2-3 days until CHO cells recovered their normal growth rate and viability. Master cell banks (MCBs) were created in which vials of cells were cryopreserved in liquid nitrogen, and assayed for sterility, mycoplasma, and adventitious agent testing at Charles River Laboratories (Malvern, PA).

Shake flask production of fusion proteins was performed at Akston’s manufacturing facility (Beverly, MA). MCB cells were first cultured for scale-up in CD OptiCHO medium containing 100 µM MSX at 37 °C prior to inoculation of fresh 10 L shake flask culture Dynamis medium (Thermo Scientific) at 37°C. Cells were fed with 2-6% volume/day Efficient Feed C+ supplement (Thermo Scientific) fortified with glucose for approximately 8 days, with a temperature shift to 32°C on Day 3. Culture medium was harvested *via* depth filtration (Pall Corporation, Port Washington, NY). Further purification was performed *via* Protein A column (MabSelect Sure, Cytiva Life Sciences, Marlborough, MA) column pre-equilibrated with phosphate buffered saline (PBS) binding buffer pH 7.4, in which column-bound Fc-fusion proteins was washed with additional binding buffer, eluted with 100 mM glycine, pH 3.5 elution buffer, and after a low pH viral inactivation step, Fc-fusion protein solution was buffer exchanged into 50 mM sodium phosphate buffer pH 7.0 using ultrafiltration-diafiltration (TengenX SIUS 30 kDa, Repligen, Waltham, MA). Fc-fusion proteins were further purified using an anion exchange chromatography step operating in flow-through mode using Q-HP resin (Cytiva). Fc-fusion protein material was again buffer exchanged into PBS (Thermo Fisher Scientific) using the same ultrafiltration-diafiltration method described above, and then filtered through a nanoporous filter (Planova® 20N, Asahi-Kasei) to accomplish viral filtration, before final 0.2 µm microfiltration (Pall Corporation).

Concentrations were confirmed *via* spectrophotometry analysis using A280 that were approximately 10 mg/mL. Batches were >98% pure Fc-fusion protein with respect to molecular aggregates measured *via* SEC-HPLC and fragments *via* capillary electrophoresis-sodium dodecyl sulfate (CE-SDS) analysis. 10 mL drug product contained 100 mg AKS-107 (1,570 nmol AKS-107), 35.5 mg sodium phosphate dibasic, EP-grade, 30.0 mg sodium phosphate monobasic, EP-grade, 87.7 mg sodium chloride, EP-grade, 2 mg polysorbate 80 (Tween-80), EP-grade, 10 mL water for injection, pH adjusted to 7.5 using hydrochloric acid or sodium hydroxide in Type I borosilicate glass vials with butyl rubber stoppers and aluminum crimped caps and stored at 2-8°C.

1. **AKS-107 binding potency to insulin-specific antibodies**

The conformational integrity of the insulin analog moiety of AKS-107 was assessed *via* binding to two anti-insulin mAbs (*i.e.*, 125mAb capture/123mAb detection were purified from hybridomas and were a gift from Dr. James Thomas) *via* a basic ELISA method (see Supplemental Methods for details) and Optical density was read *via* a microplate reader (Spectra Max 190, Molecular Devices, San Jose, CA) at 450 nm using SoftMaxPro software (V5.4, Molecular Devices). Nunc ImmunoMaxiSorp 96-well Plates (Thermo Fisher Scientific, Waltham, MA) were coated with 100 µL of 10 µg/mL 125mAb in pH 9.6 sodium bicarbonate buffer overnight at 4°C. Plates were washed 5 times with PBS containing 0.05% Tween20 (PBST; Boston Bioproducts, Milford, MA) followed by addition of 200 µL Superblock blocking buffer (Thermo Fisher) at room temperature for 1 h. Different dilutions of AKS-107 or human recombinant insulin (hrINS) (Sigma-Aldrich, Burlington, MA) were prepared in sample dilution buffer (PBST + 10% Superblock) and 100 µL was added to the 125mAb-coated plates for 1 hr at room temperature, washed 5 times with PBST, and incubated with 100 µL biotinylated 123mAb (1 µg/mL) in sample dilution buffer for 1 hr at room temperature. After washing 5 times, 100 µL of streptavidin-HRP (Abcam, Waltham, MA) in sample dilution buffer (1:15,000 dilution) was added and incubated for 45 min at room temperature in the dark. Plates were washed 5 times and incubated with 100 µL TMB substrate (Thermo Fisher) for 10-15 min and stopped with 100 µL of Stop Reagent (Thermo Fisher). Optical density was read *via* a microplate reader (Spectra Max 190, Molecular Devices, San Jose, CA) at 450 nm using SoftMaxPro software (V5.4, Molecular Devices).

The binding affinity of AKS-107 to circulating human serum IAAs was used as a proxy for binding the repertoire of insulin-reactive BCRs of B cells from which IAAs originate.  rhINS and AKS-107 preparation were evaluated for the capacity to inhibit ^125^I-labeled rhINS (Perkin Elmer, Waltham, MA) from binding IAAs in pooled serum samples from pre-diabetic IAA^+^ human subjects (obtained at the Barbara Davis Center for Diabetes Pediatric Clinic and the NIH/NIDDK Autoantibody/HLA Core Lab, Aurora, CO) *via* radioimmunoassay.  Briefly, serum was incubated overnight with ^125^I-rhINS (20,000 cpm) with and without dilution of cold rhINS or AKS-107.  IAAs bound to ^125^I-rhINS were then precipitated *via* protein-A/G Sepharose in a 96-well plate format, washed to remove unbound ^125^I-rhINS, and radioactive cpm/well were determined using a 96-well plate β counter (Perkin Elmer).

1. **AKS-107 binding affinity to insulin receptors expressed on IM-9 cells**

Human IM-9 cells (ATTC# CCL-159) naturally expressing the human insulin receptor were cultured and maintained in complete medium (RPMI-1640 containing 10% FBS) at 70-80% confluency. Cells were harvested and centrifuged at 250x*g* for 10 min and the cell pellet was washed once with HBSS, resuspended in chilled FACS buffer (HBSS/2mM EDTA/0.1% Na-azide + 2% horse serum; Sigma-Aldrich) to a concentration of 10^7^ cells/ml, and incubated on ice for 20-30 min. 50 µL of test compounds, AKS-107 and rhINS, serially diluted in chilled FACS buffer were added to 5 µL of a 10 µg/mL solution of biotin-labeled rhINS diluted in FACS buffer (final 0.5 µg/mL) in wells of a V bottom microtiter plate (Thermo Fisher), mixed, and placed on ice while 45 µL of the IM-9 cell suspension (*i.e.*, 4.5 x 10^5^) was added to each well. Cell mixtures were incubated on ice for 30 min to allow competitive binding to insulin receptors and then washed and resuspended in 50 µl of FACS buffer containing 1:200 dilution of streptavidin-phycoerythrin (PE) (Thermo Fisher), and incubated on ice for 20 min. Cells were washed, fixed with 4% paraformaldehyde, and transferred to FACS tubes for analysis *via* FACSCalibur. Cell-bound PE-rhINS was calculated as the median fluorescence intensity (MFI) per sample in which MFI values of cells with PE-rhINS in the absence of AKS-107 or unlabeled rhINS were used as the 100% binding controls. IC50 values were calculated using the non-linear *[Inhibitor] vs. response-Variable slope (four parameters)* algorithm in GraphPad Prism 10.1 software (GraphPad Software, Boston, MA).

1. **Insulin receptor phosphorylation**

U2OS cells genetically engineered to express the human insulin receptor (PathHunter® Insulin Bioassay kit, Eurofins/DiscoverX, Fremont, CA; <https://www.discoverx.com/product/pathhunter-insulin-bioassay-kit/>) were incubated with different concentrations of rhINS (positive control), AKS-130 (active insulin-Fc analog positive control), AKS-107, or human IgG mAb (Fc negative control) and kinase activity was assessed by the addition of chemiluminescence substrate (PathHunter Detection reagent cocktail; PerkinElmer Envision™ instrument). Briefly, cells were thawed, resuspended in Cell Plating Reagent 5 solution, seeded in a total volume of 80 μL (2 x 10^4^ cells) per well of a 384-well white-walled microplate, and incubated at 37 °C for 24 h. Cells were then treated with 20 µL of test molecule dilutions incubated at room temperature for 3 h. 10 μL of PathHunter Detection Reagent 1 was added to cells for 15 min at room temperature followed by addition of 40 uL Detection Reagent 2 (chemiluminescent substrate for activated receptor kinase) for 90 min in the dark at which time chemiluminescence was measured that reflects the degree of receptor kinase activation (PerkinElmer Envision™ instrument). Chemiluminescence data were processed and analyzed *via* CBIS data analysis software (ChemInnovation, San Diego, CA) and IC50 values were calculated using the non-linear *[Inhibitor] vs. response-Variable slope (four parameters)* algorithm in GraphPad Prism 10.1 software.

1. **Cytotoxicity assay of murine insulin-reactive B cells**

To assess the ability of the Fc component of AKS-107 to mediate cytotoxicity of insulin-reactive B cells, an *in vitro* assay was developed using Tg125(H+L)/NOD mouse splenocytes (the B lymphocyte compartment contains >95% insulin-binding B cells) and bone marrow-derived macrophages. Tg125(H+L)/NOD splenocytes and macrophages were isolated as previously described [43, 51]. Splenocytes (5x10^5^ cells) and macrophages (2.5x10^4^ cells) were cultured in culture medium (IMDM with 10% fetal bovine serum) in wells of sterile round-bottom 96-well tissue culture plates (Thermo Fisher) with or without AKS-107 (5-fold serial dilutions) for 72 h (200 µL total volume), washed 3 times to remove AKS-107, and cultured in 200 µL of culture medium for an additional 24 h to allow for B cell receptor turnover. Cells were then transferred to V-bottom microtiter plates, washed once with cold MACS buffer (Miltenyi Biotech, Bergisch Gladbach, Germany), resuspended in 60 µL of FACS staining medium (HBSS/0.1% Sodium Azide/4% horse serum) containing 3 µL of rhINS-biotin/streptavidin-labeled Microbeads (Miltenyi Biotech, , #130-048-101) plus 2 µL of Alexa Fluor® 488-labelled B220 mAb (1 mg/mL), and incubated on ice for 30 min. Cells were washed twice with cold-MACS buffer, resuspended in 50 µL of FACS staining buffer containing APC-labelled anti-µBead mAb for 20 minutes on ice, washed once, and fixed with 2% paraformaldehyde for flowcytometric analysis (FACSCalibur, Becton Dickinson, Franklin Lakes, NJ). AKS-107 concentration-dependent killing of insulin^+^ B cells was calculated as the percentage of insulin^+^ B cells from control cultures in which EC50 and EC90 values were calculated using the non-linear *[Agonist] vs. response-Variable slope (four parameters)* algorithm in GraphPad Prism 10.1 software.

# In vivo pharmacokinetics and pharmacodynamics in non-human primates (NHPs)

- 1. All NHP studies were performed with protocols approved by the Animal Care and Use Committee (IACUC) at Biomere (Worcester, MA) in accordance with the National Institutes of Health guide for the care and use of laboratory animals (NIH Publications No. 8023, revised 1978), and with veterinary care in accordance with the testing facility standard operating procedures and regulations outlined in the applicable sections of the Final Rules of the Animal Welfare Act regulations (9 CFR).
  2. *Single dose i.v. pharmacokinetics and blood glucose levels in NHPs*: Male, naïve cynomolgus monkeys from the testing facility colony (Biomere, Worcester, MA) were received in good health, and then housed and cared for according to Testing Facility IACUC Guidelines and SOPs. Cynomolgus monkeys were selected for the study for two reasons: (i) NHPs are a commonly used toxicology species for biologic therapeutics, including antibodies and Fc fusion proteins, and (ii) NHPs have the most species homology to the human Fc and the human FcRn receptor sequences. Cynomolgus monkeys are also particularly good choices within the group of NHP species due to their small size, thus requiring smaller quantities of compound. Animals were restrained manually and/or mechanically (cage-side and/or chair) as necessary for dose administration and blood collection procedures. NHPs (N=3 males) received an i.v. injection of test article AKS-107 at 0.4 mg/kg (6 nmol/kg) on Day 1. Animals were fasted overnight before Day 1 dosing, and before the blood glucose measurements were taken on Days 2, 3, 4, 5, 6, 7, 8, 15, 22 and 29. Blood samples for blood glucose measurement or serum for PK analysis were collected from a peripheral vein via direct needle puncture at the appropriate time points (Pre-dose and t = 15 min, 1, 2, 4, 8, 24, and 48 hrs post-dose, and Days 4, 5, 6, 7, 8, 15, 22, and 29 post dose). For each time point, the glucose level was immediately determined via glucose monitor. Once the glucose measurement was obtained, the remaining 1 mL of whole blood required for PK analysis was placed into a serum separator blood collection tube (SST) and allowed to clot at room temperature before being processed by centrifugation. The tubes were then centrifuged at 2,800-3,000 rpm for 10 min at room temperature. Each serum sample was aliquoted into 2 cryovials, snap-frozen on dry ice, and stored at -20 °C until needed.
  3. *PK Analysis of AKS-107 in NHP serum*: Two types of sandwich ELISAs were performed to measure the concentration of AKS-107 in serum samples collected from cynomolgus monkeys. The first assay was designed to first capture the AKS-107 Insulin-Hu Fc fusion protein therapeutic via its Fc fragment using a selective anti-human Fc antibody that does not cross-react with cyno IgG Fc, and then to detect the bound AKS-107 using an anti-human IgG-HRP secondary antibody. The second ELISA was designed to first capture the AKS-107 at a particular epitope of the insulin portion of the molecule using the anti-human insulin antibody, mAb125, and then to detect the bound AKS-107 using a biotinylated anti-insulin antibody, mAb123, that binds to a different epitope of the insulin portion of the AKS-107 molecule (as described above in *Section 3*). For the huFc capture/huFc Detection ELISA, Nunc maxisorp ELISA plates were coated with Neutravidin (Thermofisher) at 10 µg/mL in pH 9.6 NaCarb-Bicarb coating buffer overnight at 4°C. Plates were washed 5x with PBST (PBS+0.05%Tween-20) buffer and blocked with Superblock blocking buffer for 1 h at room temperature. CaptureSelect biotinylated anti-human Fc (Thermofisher) was then added at 5 µg/mL diluted in PBST/10% Superblock dilution buffer for 1 hr at room temperature for selective binding of the human Fc-containing fusion protein compounds in cyno serum samples. Cyno serum samples were diluted from 1:20 to 1:100 in sample dilution buffer (PBST + 10% Superblock + 20% Horse Serum). AKS-107 compound was also diluted in sample dilution buffer containing a specified concentration of normal cyno serum from 600 ng/ml to 0.82 ng/ml in 1:3 serial dilutions for the standard curve (e.g. standards prepared in sample dilution buffer with 5% normal cyno serum were used for 1:20 diluted samples). Standards and diluted serum samples were added to the neutravidin-biotinylated anti-human Fc coated plates and incubated for 1 hr at room temperature. Plates were washed 5*x* with PBST (PBS+0.05%Tween20) buffer and captured AKS-107 compound was detected using Goat anti-human IgG-Fc-HRP (Bethyl Laboratory) secondary antibody 1:15000 diluted in PBST/10% Superblock dilution buffer and incubated for 45 minutes at room temperature in the dark. Finally, plates were washed 5× with PBST buffer and 1*x* wash with dH_2_O, followed by incubation with TMB substrate at 100 µL/well for 10-15 min and stopped with 100 µL/well of ELISA stop reagent. Plates were read in a microplate reader at 450 nm using SoftMaxPro software, and AKS-107 values in the samples were calculated by interpolation on a 4-PL curve for each plate.
