# Supplementary material for: An antigen-specific immunotherapeutic, AKS-107, deletes insulin-specific B cells and prevents murine autoimmune diabetes
Source: Front Immunol. 2024 Mar 7;15:1367514. doi: 10.3389/fimmu.2024.1367514 (PMC10954819; doi:10.3389/fimmu.2024.1367514)
Supplement: Supplementary file 2 [file Table_1.docx]

**Supplementary Table S1:** amino acid sequences of insulin analog Fc-fusion molecules

| **hIgG1 Fc-Insulin analog Fusion Protein** | **Full Amino Acid Sequence (Insulin Analog Portion in red text)** |
| --- | --- |
| **AKS-107** | **FVNQHLCGSHLVEALALVCGERGFFYTPKAAKGIVEQCCTSICSLYQLENYCN**GGGGAGGGGDKTHTCPPCPAPELLGGPSVFLFPPKPKDTLMISRTPEVTCVVVDVSHEDPEVKFNWYVDGVEVHNAKTKPREEQYNSTYRVVSVLTVLHQDWLNGKEYKCKVSNKALPAPIEKTISKAKGQPREPQVYTLPPSRDELTKNQVSLTCLVKGFYPSDIAVEWESNGQPENNYKTTPPVLDSDGSFFLYSKLTVDKSRWQQGNVFSCSVMHEALHNHYTQKSLSLSPG |
| **AKS-130** | **FVNQHLCGSDLVEALALVCGERGFFYTDPTGGGPRRGIVEQCCHSICSLYQLENYCN**GGGGAGGGGDKTHTCPPCPAPELLGGPSVFLFPPKPKDTLMISRTPEVTCVVVDVSHEDPEVKFNWYVDGVEVHNAKTKPREEQYNSTYRVVSVLTVLHQDWLNGKEYKCKVSNKALPAPIEKTISKAKGQPREPQVYTLPPSRDELTKNQVSLTCLVKGFYPSDIAVEWESNGQPENNYKTTPPVLDSDGSFFLYSKLTVDKSRWQQGNVFSCSVMHEALHNHYTQKSLSLSPG |

**Supplemental Figure S1**. AKS-107 PK and blood glucose in non-human primates. The lack of insulin receptor activation by AKS-107 was determined in vivo in cynomolgus monkeys after one (A) or three (B) 0.4 mg/kg i.v. injections of AKS-107 or the human IgG Fc mAb control, rituximab, and measuring fasting blood glucose and serum levels of AKS-107 (via ELISA) during 28 days. AKS-07 serum t_1/2_ was calculated via the *Exponential-One Phase Decay* algorithm in GraphPad Prism 10.1 software. (see *Supplemental Methods* for details)

**Supplemental Figure S2**: AKS-107 binding to human Fc receptor isotypes. Recombinant human Fc receptors (FcR; including complement 1q, C1q) were bound to plastic ELISA plates and different forms of AKS-107 (standard, deglycosylated, or Fab’2 fragments without Fc moiety) or the human Fc positive control mAb, rituximab, were added to plates and allowed to the bind FcRs. After washing, Fc-bound molecules were detected via labelled anti-human IgG. Data represent the % maximal Rituximab binding.
